# Proximal femoral derotation osteotomy for idiopathic excessive femoral anteversion and intoeing gait

**DOI:** 10.1051/sicotj/2017033

**Published:** 2017-07-04

**Authors:** Gohar Naqvi, Kuldeep Stohr, Andreas Rehm

**Affiliations:** 1 Department of Orthopaedics Surgery, Addenbrookes Hospital Hills Rd Cambridge CB2 0QQ UK

**Keywords:** Intoeing, Femoral anteversion, Femoral osteotomy

## Abstract

*Aim*: The purpose of this study is to assess the symptoms caused by excessive femoral anteversion and the outcomes of femoral derotation osteotomy.

*Methods*: We reviewed data on patients who underwent proximal femoral derotation osteotomy for symptomatic intoeing gait caused by femoral anteversion. Only symptomatic patients were considered for corrective derotation osteotomy. Degree of femoral anteversion was confirmed on computed tomography (CT) scan.

*Results*: Thirty-five extremities were operated in 21 patients with an average age of 13.3 (8–18) years. Mean follow-up was 16 months (6–36 months). Mean femoral anteversion angle was 40.8° (28°–53°). External rotation of extended hips improved significantly, from 30° to 51.8° (*p* < 0.0001). Mean foot progressing angle improved from 15.2° internally rotated preoperatively to 7.7° externally rotated. Intoeing completely resolved in all except two patients. Thirteen out of 21 children complained about tripping and frequent falling while running and playing sports, eight patients had hip pain while 13 children had knee pain preoperatively. Tripping, falling and hip pain resolved in all patients postoperatively, while three patients whose primary complaint was knee pain failed to improve postsurgery. Eighteen of the 21 parents were satisfied with the decision to perform surgical correction.

*Conclusion*: Excessive femoral anteversion can present with unexplained hip or knee pain refractory to conservative treatments. Careful assessment of lower limb malalignment is a valuable tool in such circumstances and derotation proximal femoral osteotomy can certainly be a procedure of choice in carefully selected cases.

## Introduction

Intoeing gait is frequently seen in developing children and is a major reason for referral to paediatric orthopaedic surgeons [1]. Intoeing is defined as a gait with internally rotated foot progression angle [2, 3]. Common causes include metatarsus adductus, excessive internal tibial torsion and increased femoral anteversion [4]. In most cases, the gait pattern and foot progression angle gradually improve with age. However, excessive femoral anteversion remains the most common cause of persistent intoeing gait in adolescent children and may disrupt the gait function [4]. Traditionally, a more conservative approach has been recommended towards the treatment of excessive femoral anteversion owing to its potential to correct spontaneously and high risk of complications, while others consider it only a cosmetic problem [5–7]. These patients often present with hip and knee symptoms along with the complaint of intoeing, clumsiness of gait and frequent falling. Recent literature has confirmed the association of excessive femoral anteversion with femoroacetabular impingement, labral tear [8–10] and anterior knee pain due to patellofemoral malalignment [11]. In patients with persistent symptoms despite extensive physiotherapy and rehabilitation, surgical correction can be performed using derotation femoral osteotomy at different levels. Although more commonly reported for cerebral palsy patients, literature is limited regarding outcomes in idiopathic cases.

## Aim

We aim to present a case series of paediatric patients with symptomatic excessive femoral anteversion treated in our institution with proximal femoral derotation osteotomy.

## Methods

We reviewed clinical and radiological data on patients who underwent proximal femoral derotation osteotomy between April 2004 and August 2014. Patients were included in this cohort only if the surgery was performed for symptomatic intoeing gait caused by excessive femoral anteversion. All patients presenting to clinic with lower limb complaints were assessed by one of the two fellowship trained paediatric orthopaedic surgeons. Presenting complaints were documented in patient’s notes, including hip, thigh or knee pain, clumsiness of gait or frequent falling. Rotational profile of lower limbs was carefully assessed according to Staheli’s method to ascertain the cause of intoeing gait. Excessive femoral anteversion was suspected clinically when the internal rotation of hips in extension measured significantly greater than external rotation. Patients with excessive femoral anteversion with symptoms of hip, or knee pain or frequent falls were considered for corrective derotation osteotomy only if symptoms persisted following extensive physiotherapy and no other convincing cause was found for pain. The degree of femoral anteversion was confirmed on computed tomography (CT) scan, by measuring an angle between the axis of femoral neck and a line drawn along the posterior aspects of both femoral condyles.

### Exclusion criteria

Children younger than eight years were observed for spontaneous correction. Patients with asymptomatic femoral anteversion were excluded from surgical intervention, irrespective of age and degree of femoral anteversion.

### Surgery and follow-up

**Figure 1.**
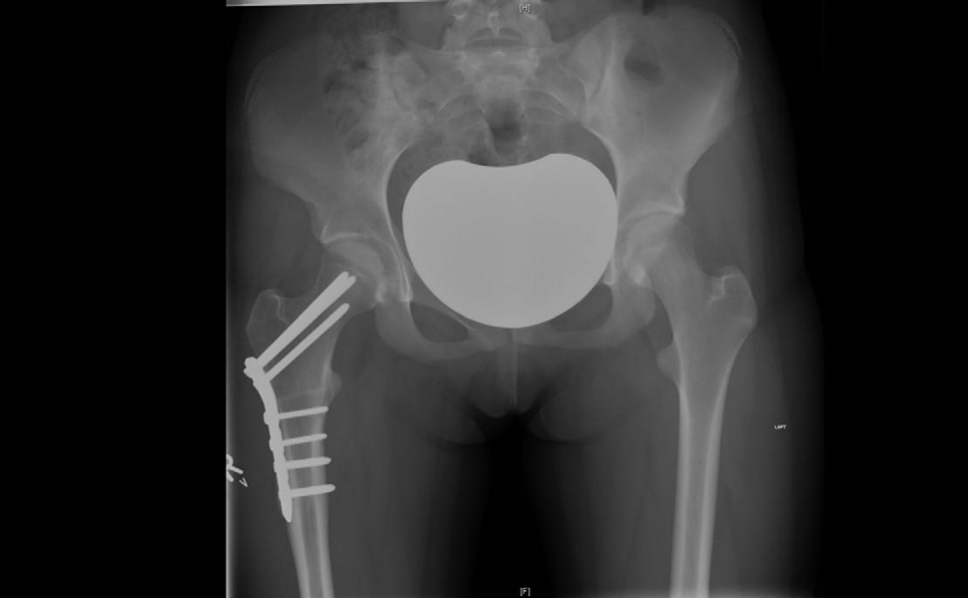
Radiograph of pelvis and hips, at three months postderotation proximal femoral osteotomy on right side.

Patients were operated by two paediatric orthopaedic surgeons using lateral sub-vastus approach and subtrochanteric osteotomy that was fixed with either fixed angle blade plate earlier in this cohort or locking compression plate (LCP) proximal femoral locking plate. Two k-wires were inserted, one proximally in the greater trochanter and the other in femoral shaft distal to the proposed location of plate fixation, at an approximate angle between each other, corresponding to the desired correction angle so that after osteotomy, the femur can be rotated to align both wires parallel to achieve the desired correction of femoral rotation. The approximate angle of correction was determined preoperatively by the operating surgeon, taking into account the measured anteversion angle and the difference in internal and external rotation of hips in extension. Furthermore, a longitudinal mark was made on the femur with a saw blade in the subtrochanteric area to retain the femoral alignment marker in case of loosening k-wire orientation and also as a second marker for correction of rotation. Jig wires were placed in the femoral neck for proximal screws, avoiding the trochanteric apophysis and femoral capital epiphysis. A transverse subtrochanteric osteotomy was performed with an oscillating saw and fixed with a proximal femoral plate after externally rotating the distal femur according to a previously decided correction angle. Patients were allowed to partially weight bear as tolerated and were followed in clinic at three weeks, three months and six months on average (Figure 1).

### Variables

Data were collected regarding demographics of patients, presenting symptoms and physical examination, including foot progression angle, internal and external rotation of hips. Radiographs and CT scans were assessed and the femoral anteversion angle was recorded for all patients. Data regarding approximate correction angles were collected from operative notes.

In the absence of a specific validated outcome instrument, postoperatively, data were recorded regarding patient’s/parent’s satisfaction, hip pain, knee pain, gait problem and frequent falling. Resolution of preoperative symptoms was considered as the primary end point. Time to radiological healing, time to full weight bearing and any complications were also recorded.

### Statistical analysis

Continuous data is presented as mean value with standard deviation. Paired *t* test was used to compare mean values preoperatively and postoperatively. Categorical data were analysed using Fisher’s exact test with *p* value <0.05 to be considered as significant.

## Results

Thirty-five extremities were operated in 21 patients with an average age of 13.3 (8–18) years. Six were male and 15 were female. Mean follow-up was 16 months (6–36 months). Mean femoral anteversion angle was 40.8° (28°–53°) (Table 1).

**Table 1:** Descriptive data on patients with proximal femoral derotation osteotomy for excessive femoral anteversion.

No. of patients	21
Male	6
Female	15
No. of limbs operated	35
Average age	13.3 (8–18) yr
Reason for attendance	
Intoeing	19
Hip pain	8
Knee pain	13
Tripping/falling	13
Foot progression angle (degree)	−15.2 (−35 to 0)
Average hip rotation (degree)	
Internal	71.2 (20–90)
External	30 (0–50)
Average femoral anteversion on CT	40.8 (28–53)
Average correction angle (intra-operative)	23 (15–25)
Average healing time for osteotomy (weeks)	15 (12–20)
Primary complaint resolved (limbs)	30/35 (85.7%)
Satisfaction (pts.)	18/21 (85.7%)
Complications (pts.)	5/21 (23.8%)

### Physical examination

Changes in rotational profile of lower limb examination between pre- and postoperative assessment are summarized in Table 2. External rotation of extended hips improved significantly, from 30° to 51.8° (*p* < 0.0001). Mean foot progressing angle improved from 15.2° internally rotated preoperatively to 7.7° externally rotated when assessed at follow-up. Intoeing was obvious on examination in 19 out of 21 patients preoperatively which resolved in 17 patients postoperatively while mild residual intoeing remained in two patients (Table 3).

**Table 2.** Physical examination data pre- and postderotation osteotomy.

Parameters	Preoperative (degree)	Postoperative (degree)	Paired *t* test
Hip internal rotation	71.2 ± 12.9 (20–90)	47.4 ± 8.8 (30–60)	<0.0001
Hip external rotation	30 ± 13.5 (0–50)	51.8 ± 11.2 (30–70)	<0.0001
Foot progression angle	−15.2 ± 9.7 (−35–0)	7.7 ± 6.1 (−5–15)	<0.0001

Values are given as means ± standard deviation (range). Foot progression angle with negative value indicates internal rotation while positive value indicates external rotation.

**Table 3.** Patient/Parent reported outcome variables.

Problem	Preoperative	Postoperative	*p*
Intoeing	19	2	<0.0001
Tripping/Falling	13	0	<0.0001
Hip pain	8	0	0.003
Knee pain	13	3	0.003
Satisfied	NA	18	

### Patient/parent reported outcomes

Table 3 summarizes the functional outcomes as reported by patients and parents preoperatively and at the latest follow-up. Thirteen out of 21 children complained about tripping and frequent falling while running and playing sports, eight patients had hip pain while 13 children had knee pain preoperatively. Tripping, falling and hip pain resolved in all patients postoperatively. Ten out of 13 complaints of knee pain were resolved while three patients whose primary complaint was knee pain failed to improve postsurgery. Eighteen of the 21 parents were satisfied with the decision to perform surgery.

### Complications

Five patients suffered complications. Three patients had persistent knee pain and in two cases, it was even worse than preoperative pain. Those two patients were reassessed further and had to undergo tibial osteotomy to correct malalignment in tibia. Both patients improved clinically after tibial osteotomy. One patient shows loosening of the metalwork but the osteotomy was healed and the patient remained asymptomatic at final follow-up. Only one patient had non-union of femoral osteotomy leading to fatigue failure of the plate, requiring revision surgery. Her osteotomy healed satisfactorily post-revision surgery and the patient was finally symptom free.

## Discussion

Intoeing gait is a common reason for attendance at paediatric orthopaedic clinic as is patient and parental anxiety due to cosmetic as well as functional reasons. It is characterized by a gait pattern with an internally rotated foot progression angle. Thackeray and Beeson [12] reported the prevalence of up to 30% in children aged six years or younger, which decreases to 7% in children aged nine years and older, signifying the spontaneous correction during the growth. Intoeing is caused by one of the three deformities, including metatarsus adductus, internal tibial torsion and femoral anteversion. Internal tibial torsion is the most common cause in children between three and six years of age while excessive femoral anteversion is common in children 6–10 years of age. The natural course of femoral anteversion has been demonstrated by Staheli et al. [3] and Fabry et al. [13] showing regression of angle during childhood but little or no change after eight years of age. Intoeing sometimes continues to improve after eight years of age mainly due to compensatory lateral tibial torsion without change in femoral anteversion [14]. This can lead to rotational mal-alignment of lower limb often referred to as “miserable malalignment syndrome” where increased femoral anteversion is compensated with increased lateral tibial torsion. Although foot progression angle is improved in such cases it develops patellar maltracking, instability and anterior knee pain due to increased Q angle.

In contradiction to the common belief that intoeing is only a cosmetic problem and should be managed nonoperatively, there are functional implications of rotational malalignment of the lower limb, especially if it is limiting their involvement in sport, as well as psychological effects due to cosmesis. Stevens et al. [11] published the success of femoral torsional correction in patients who had developed anterior knee pain and/or patellar instability before skeletal maturity and had failed to improve after one or more knee surgeries. Recent literature has also implicated increased femoral anteversion as a cause for femoroacetabular impingement and labral tear [8, 9, 15].

Assessment of children presenting with unexplained hip thigh and anterior knee pain should include a rotational profile of the lower limb irrespective of gait pattern. Staheli’s rotational profile is the most commonly referred to standard of assessment. It includes foot progression angle, thigh foot angle, comparison of internal and external rotation of extended hips. It is designed to differentiate clinically between femoral anteversion, tibial torsion and metatarsus adductus. Suspected cases of increased femoral anteversion can be further assessed with CT scan to measure the angle of femoral anteversion and tibial torsion. All patients should have undergone an extensive course of physiotherapy including posture and gait exercises, quadriceps and vastus medialis oblicuus strengthening exercises. Patients younger than eight years of age should be observed as spontaneous regression of increased femoral anteversion is possible in most cases. Surgery should only be limited to patients above eight years of age with increased femoral anteversion and symptoms that persist despite extensive physiotherapy.

All patients in our series were older than 10 years of age, except for one girl aged eight years. She was operated upon after a long discussion relating to frequent falls secondary to intoeing gait which caused anxiety as she was not able to take part in physical activities at school. None of the patients was operated upon purely for cosmetic reason. Their primary complaint was monitored and documented in the postoperative period in order to measure of the success of the procedure. Two patients had to undergo further surgery of the tibia as their knee pain got worse after femoral derotation osteotomy. This signifies the importance of careful assessment of rotational as well as the angular alignment of the whole of the lower limb as sometime there is compensatory increased external tibial torsion due to the increased femoral anteversion, termed as “miserable alignment”. The correction of femoral anteversion alone in such cases can have a detrimental effect on external tibial torsion and patellar tracking made worse. Double osteotomy would be an option in carefully selected cases.

Although this study shows favourable clinical outcomes of proximal femoral derotation osteotomy, it is limited by lack of a validated scoring system addressing the problem and also lack of objective measurements of postoperative femoral anteversion angles with CT scans. It would have been a useful adjunct to have gait lab analysis of pre- and post-corrective osteotomy and should be considered for future studies.

## Conclusion

Excessive femoral anteversion is a common cause of intoeing gait in children. Intoeing and increased femoral anteversion can improve with growth in most children but little or no improvement has been reported after eight years of age. Children with persistently increased femoral anteversion can present with unexplained hip or knee pain refractory to conservative treatments or frequent falling and the inability to take part in physical activities effectively, leading to psychological implications. Careful assessment of lower limb malalignment is a valuable tool in such circumstances and derotation proximal femoral osteotomy can certainly be a procedure of choice in carefully selected cases.

## Conflict of interest

All authors (GN, KS, AR) certify that he or she has no financial conflict of interest (e.g., consultancies, stock ownership, equity interest, patent/licensing arrangements, etc.) in connection with this article.
